# A cholera outbreak in Alborz Province, Iran: a matched case-control study

**DOI:** 10.4178/epih.e2016018

**Published:** 2016-05-14

**Authors:** Ghobad Moradi, Mohammad Aziz Rasouli, Parvin Mohammadi, Elham Elahi, Hojatollah Barati

**Affiliations:** 1 Social Determinants of Health Research Center, Kurdistan University of Medical Sciences, Sanandaj, Iran; 2 Department of Epidemiology and Biostatistics, Kurdistan University of Medical Sciences, Sanandaj, Iran; 3 Department of Nursing and Midwifery, Sanandaj Branch, Islamic Azad University, Sanandaj, Iran; 4 Health Education and Health Promotion, School of Public Health, Shahid Beheshti University of Medical Sciences, Tehran, Iran; 5 Health Department, Alborz University of Medical Sciences, Karaj, Iran

**Keywords:** Cholera, Disease outbreaks, Case-control studies, Iran

## Abstract

**OBJECTIVES:**

A total of 229 confirmed cholera cases were reported in Alborz Province during an outbreak that lasted from June 2011 to August 2011. This study aimed to identify potential sources of transmission in order to determine suitable interventions in similar outbreaks. In other words, the lessons learned from this retrospective study can be utilized to manage future similar outbreaks.

**METHODS:**

An age-matched and sex-matched case-control study was conducted during the outbreak. For each case, two control subjects were selected from the neighborhood. A case of cholera was defined as a bacteriologically confirmed case with signs and symptoms of cholera. This study was conducted from June 14, 2011 through August 23, 2011. The data were analyzed by calculating odds ratios (ORs) using the logistic regression method.

**RESULTS:**

In this outbreak, 229 confirmed cholera cases were diagnosed. The following risk factors were found to be associated with cholera: consumption of unrefrigerated leftover food (OR, 3.05; 95% confidence interval [CI], 1.72 to 5.41), consumption of vegetables and fruits in the previous three days (OR, 2.75; 95% CI, 1.95 to 3.89), and a history of traveling in the previous five days (OR, 5.31; 95% CI, 2.21 to 9.72).

**CONCLUSIONS:**

Consumption of vegetables and fruits has remained an unresolved risk factor in cholera outbreaks in Iran in recent years. In order to reduce the risk of cholera, sanitary standards for fruits and vegetables should be observed at all points from production to consumption, the population should be educated regarding hygienic food storage during outbreaks, and sanitary standards should be maintained when traveling during cholera outbreaks.

## INTRODUCTION

Cholera is an infectious disease that presents with acute watery diarrhea and is caused by the *Vibrio cholerae* serogroups O_1_ and O_139_. It has caused large epidemics in many countries worldwide [1,2]. This disease poses a serious threat to public health, particularly in countries where people have limited access to safe water and sanitary toilets [3].

In 2007, a total of 177,963 cases were reported to the World Health Organization, as well as 4,031 deaths; this figure reflected a 25% increase in incidence compared to 2006 [4,5]. It is estimated that approximately three to five million people are annually infected with cholera, and that cholera causes 100,000 to 120,000 deaths worldwide. The fatality rate from cholera is highest in Africa, whereas case fatality rates were reported to be less than 1% in Asia in a 2002 study [6]. In Iran, a large cholera outbreak occurred in 1999, in which more than 10,000 people were infected and 109 people died. In another large out break in Iran in 2006, 1,150 confirmed cholera cases occurred, resulting in 11 deaths [7,8]. The risk factors for cholera include poverty, underdevelopment, population density, low education levels, travel to high-risk areas, consumption of leftover food, and consumption of unsafe fruits and vegetables [9].

Other risk factors for cholera outbreaks are posed by sudden changes in sanitary behavior involving water and toilet use [10, 11]. Natural disasters such as floods can lead to the deterioration of sanitary conditions and inadequate health services. Sudden population movements and the need to find accommodation for refugees may likewise affect water and health resources. It is very important to be aware of the modes of transmission of a disease during outbreaks, and these modes of transmission may vary according to regionally specific circumstances [12,13].

Cholera outbreaks have occurred with some frequency in Alborz Province due to its specific circumstances. The aim of this study, which was conducted concurrently with the cholera outbreak in Alborz Province in 2011, was to determine the risk factors associated with cholera infection in order to control the outbreak. In other words, the lessons learned from this retrospective study can be utilized to manage future similar outbreaks.

## MATERIALS AND METHODS

In order to determine the risk factors associated with cholera, we conducted an age-matched and sex-matched case-control study. The study was conducted from June 14, to August 23, 2011. A questionnaire was developed and administrated by trained interviewers, including questions dealing with demographic characteristics, symptoms, and potential exposure through food, water, personal hygiene, and sanitation. A case was defined as any person suffering from watery diarrhea whose illness was confirmed as cholera based on a laboratory analysis.

Patients with cholera were interviewed after laboratory confirmation of the infection. Interviewers visited the place of residence of each case and selected controls from the same neighborhood. Every case was matched with two controls of a similar age group (within five years) and of the same sex. Controls and their household members were required to have no history of diarrhea. Information was collected on 229 cases and 458 controls. Exposure-related variables were included in the unconditional logistic regression analysis used in the multivariate model if they displayed a p-value <0.1 in the univariate analysis. We calculated the odds ratios (ORs) and 95% confidence intervals (CIs) associated with the independent variables. We used Stata version 12.0 (StataCorp, College Station, TX, USA) to analyze the data, and employed Clopper-Pearson binomial CIs. Data were analyzed using SPSS version 20.0 (IBM Corp., Armonk, NY, USA). Stata software was used to analyze the data via regression test, and other types of statistical analysis were performed using SPSS software.

## RESULTS

**Figure 1.**
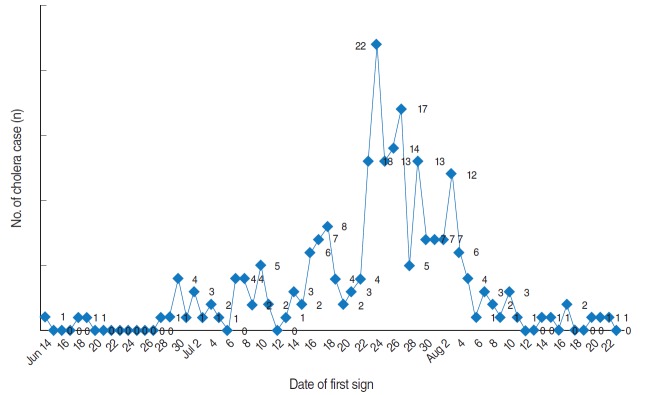
Epidemic curve of cholera outbreak in Alborz Province, 2011.

The first case of cholera was reported on June 14, 2011. According to the available data, the outbreak started on June 14, 2011, and continued to August 23, 2011. The peak of the outbreak was on July 23, with 22 cases reported that day. A total of 229 cases were positive for the El Tor biotype, and all cases were of the Ogawa serotype. Figure 1 shows the epidemic curve for Alborz Province.

The mean age of both cases and controls was 37 years (range, 1 to 86 years). A total of 67.7% of the cases were treated in the hospital. The demographic characteristics of cases and controls are presented in Table 1.

The patients with cholera reported the following symptoms: vomiting (64.6%), nausea (56.8%), abdominal pain (24.5%), headache (24.5%), myalgia (33.6%), watery diarrhea (87.3%), and bloody diarrhea (1.3%). Of all the patients with cholera, 32.3% received outpatient services (Table 2).

An updated chlorination system had been installed in the water distribution system supplying the urban area of Alborz Province, and during the epidemic, the residual chlorine in both urban and rural areas was maintained at 0.8 ppm, preventing cholera dissemination through the water supply system.

No significant association was observed in the univariate analysis between cholera and contact with diarrhea over the previous five days; in fact, this variable was found to be associated with a lower risk of cholera (OR, 1.01; 95% CI, 0.66 to 1.54); the calculated OR did not suggest a strong relationship. In addition, as shown in Table 3, no association was found between cholera and consuming food purchased from street vendors (OR, 0.92; 95% CI, 0.52 to 1.62).

In the multivariate analysis, the following risk factors for cholera infection were identified: consumption of vegetables and fruits in the previous three days (OR, 2.75; 95% CI, 1.95 to 3.89), consumption of unrefrigerated leftover food (OR, 3.05; 95% CI, 1.72 to 5.41), and a history of travel in the previous five days (OR, 5.31; 95% CI, 2.21 to 9.72) (Table 3).

## DISCUSSION

According to this case-control study, the risk factors for cholera infection were as follows: consumption of vegetables and fruits in the previous three days, consumption of unrefrigerated leftover food, and a history of travel in the previous five days.

These results are consistent with those that have been reported by other studies [1,7]. The majority of outbreaks in Iran have occurred in the warm seasons and the summer [1].

No relationship was found between cholera infection and contact with other cases of diarrhea in the previous five days (OR, 1.01; 95% CI, 0.66 to 1.54). Limited evidence exists regarding the role of family contacts in the transmission of infections, although some studies have found that the presence of a case at home is a risk factor [14,15] and that family members can spread cholera via contact with food and water at home [16]. A previous study found that contact with a person with diarrhea had a significant association with cholera infection [17].

Our multivariate analysis showed that vegetable and fruit consumption was a factor affecting the spread of the disease (OR, 2.75; 95% CI, 1.95 to 3.89). It also showed that patients were exposed to the disease 2.75 times more than the control group. A case-control study of a cholera outbreak in Zambia showed that the consumption of raw vegetables was significantly associated with cholera [12]. In a study carried out by Eshrati et al. [7] of an outbreak in Arak, Iran in 2005, a total of 16 patients and 32 controls were evaluated using a similar method. According to the results of that study, vegetable consumption was found to be an important risk factor for the transmission of the disease. In a case-control study conducted by Barati et al. [1] in 2008, fruit and vegetable consumption was reported to be a factor affecting the incidence of the disease. Moreover, in a study conducted by Rahbar et al. [13], the consumption of raw vegetables irrigated with wastewater was reported to be a risk factor. In another study [10,18] a significant association was observed between the consumption of vegetables and cholera, which is consistent with our study. The appropriate and timely sampling of vegetables and the proper administration of perchlorate powder to disinfect vegetables may play an important role in preventing cholera.

No significant association was found between cholera infection and purchasing food from street vendors or restaurants (OR, 0.92; 95% CI, 0.52 to 1.62). Likewise, Eshrati et al. [7] found no significant association between the consumption of commercially prepared food and cholera. Mahdavi et al. [19] similarly found no significant associations between food consumption in food stalls and the disease. Moreover, Nguyen et al. [18] performed a multivariable analysis and showed that food and drink sold by street vendors were not significantly associated with cholera. However, the consumption of food prepared from commercial vendors was reported to be a risk factor for cholera infection in outbreaks in Uganda and Haiti [20,21]. In many other outbreaks, the consumption of drinks and foods purchased from street vendors has been identified as an important risk factor [22,23]. Street food vendors represent a possible method of transmitting cholera throughout the population, as suggested by Loharikar et al. [24]. Items sold by vendors are most likely to be contaminated through the environment and by poor handling. Epidemiological evidence from Zambia showed that contaminated food was a major path of transmission of cholera during an outbreak [12]. Therefore, the most effective ways to prevent transmission of cholera are the following strategies: maintaining effective hygiene standards regarding food from production to distribution and consumption, disinfecting vegetables and fruits, and education.

The other risk factor identified in this outbreak was the consumption of unrefrigerated leftover food, which had a strong relationship with cholera (OR, 3.05; 95% CI, 1.72 to 5.41). This finding is consistent with that reported by Izadi et al. [25]. Such a relationship has been also reported in outbreaks in other countries, and in fact, a delay between cooking and eating food contaminated with *V. cholerae* is necessary for disease to occur [26, 27].

In this study, a significant association was found between a history of travel in the previous five days and cholera infection (OR, 5.31; 95% CI, 2.21 to 9.72). Compared with the controls, cholera cases have been previously found to have traveled more in the prior two weeks (p<0.05) [28]. A number of demographic and socioeconomic factors, including age, sex, nutritional status, social status, economic status, and history of travelling abroad, are also known to play a crucial role in susceptibility to choleragenic *V. cholerae*. Poor sanitation and minimal access to health facilities during travel, as well as purchasing food from street vendors or restaurants, are important factors contributing to cholera infection. It has become clear that good sanitation and hygienic practices while traveling can largely prevent infection with the disease [29,30].

The water supply, distribution, and chlorination system of Alborz Province prevents cholera dissemination through chlorination and daily chlorination monitoring during cholera outbreaks. In recent years, most of the reported outbreaks have not occurred due to problems with the water supply.

In conclusion, the consumption of fruits and vegetables has remained an unresolved risk factor for cholera outbreaks in Iran in recent years. In order to reduce the risk of cholera, sanitary standards should be observed for fruits and vegetables from the time of production to the time of consumption, individuals should be educated regarding about sanitary food storage during outbreaks, and sanitary standards should be observed during trips taken during cholera outbreaks.

Similarly to other case-control studies, this study had some limitations. It was conducted during the outbreak and had not been designed in advance; hence, the data might have not been collected comprehensively.

## Figures and Tables

**Table 1.** Demographic characteristics of cases and controls in the 2011 cholera outbreak in Alborz Province, Iran

Variable		Cases (n=229)	Controls (n=458)	p-value^1^
Sex	Male	102 (45)	203 (44)	0.95
	Female	127 (55)	255 (55)	
Age (yr)	<10	9 (4)	16 (4)	0.38
	10-19	14 (6)	23 (5)	
	20-39	117 (51)	213 (46)	
	40-59	68 (30)	173 (38)	
	≥60	21 (9)	34 (7)	
Nationality	Iranian	222 (97)	450 (98)	0.26
	Afghan	7 (3)	8 (2)	
Residence	Urban	215 (94)	430 (94)	1.00
	Rural	14 (6)	28 (6)	
Marital status	Single	58 (25)	121 (26)	0.75
	Married	171 (75)	337 (73)	
Village of residence	Villages with a health care center (Health House)	79 (11)	78 (22)	1.00
	Villages without a health care center (Health House)	21 (3)	21 (6)	

Values are presented as number (%).

1 The proportions of cases and controls were tested using the chi-square test.

**Table 2.** Frequency of clinical signs, severity of diarrhea, and type of treatment of patients with cholera

Sign	n (%)	95% CI of %
Vomiting	148 (64.6)	(58.3, 70.8)
Nausea	130 (56.8)	(50.3, 63.2)
Fever	51 (22.3)	(16.8, 27.7)
Abdominal pain	56 (24.5)	(52.5, 65.3)
Headache	56 (24.5)	(18.8, 30.0)
Myalgia	77 (33.6)	(27.4, 39.7)
Severity of diarrhea		
Low	35 (15.3)	(9.2, 21.4)
Moderate	73 (31.9)	(25.4, 37.6)
Severe	121 (52.8)	(46.8, 58.3)
Type of treatment		
Outpatient	74 (32.3)	(26.4, 38.7)
Hospitalization	155 (67.7)	(61.2, 73.5)

CI, confidence interval.

**Table 3.** Analysis for selected potential risk factors of cholera infection

Variable	Cases	Controls	Crude	p-value	Adjusted^1^	p-value
Contact with other cases of diarrhea in the past five days	25 (10.9)	21 (4.6)	1.01 (0.66, 1.54)	0.94	-	-
Vegetable and fruit consumption in the past three days	155 (67.6)	185 (40.3)	3.09 (2.21, 4.31)	< 0.001	2.75 (1.95, 3.89)	<0.001
Purchasing food from street vendors or restaurants	19 (8.2)	41 (8.9)	0.92 (0.52, 1.62)	0.77	-	-
Consumption of unrefrigerated leftover food	40 (17.4)	22 (4.8)	4.19 (2.42, 7.25)	< 0.001	3.05 (1.72, 5.41)	<0.001
History of traveling in the past five days	61 (29.7)	28 (6.1)	5.75 (3.44, 9.02)	< 0.001	5.31 (2.21, 9.72)	<0.001

Values are presented as number (%) or odds ratio (95% confidence interval).

1 Adjusted according to age and sex.
